# Nonfatal Drug and Polydrug Overdoses Treated in Emergency Departments — 29 States, 2018–2019

**DOI:** 10.15585/mmwr.mm6934a1

**Published:** 2020-08-28

**Authors:** Stephen Liu, Lawrence Scholl, Brooke Hoots, Puja Seth

**Affiliations:** 1 Division of Overdose Prevention, National Center for Injury Prevention and Control, CDC.

The U.S. drug overdose epidemic continues to cause substantial morbidity and mortality. In 2017, 967,615 nonfatal drug overdoses were treated in emergency departments (EDs), a 4.3% increase from 2016 in all overdoses and a 3.1% increase in opioid-involved overdoses (*1*). During 2017 and 2018, syndromic surveillance revealed that 37.2% of overdoses treated in EDs in 18 states involved multiple drugs (*2*). To describe changes in rates and proportions of suspected nonfatal drug and polydrug overdoses treated in EDs, CDC analyzed syndromic surveillance data from 2018 to 2019 in 29 states. Rates of overdoses involving opioids, cocaine, and amphetamines increased 9.7%, 11.0%, and 18.3%, respectively, and the rate of benzodiazepine-involved overdoses decreased 3.0%. Overdoses co-involving opioids and amphetamines increased from 2018 to 2019, overall, in both sexes, and in most age groups. In 2019, 23.6%, 17.1%, and 18.7% of overdoses involving cocaine, amphetamine, and benzodiazepines, respectively, also involved opioids. Expanding overdose prevention, treatment, and response efforts is needed to reduce the number of drug and polydrug overdoses. This includes linkage into treatment, harm reduction services, and community-based programs for persons who use drugs; expanding overdose prevention efforts, including increased naloxone provision, to persons who use stimulants; addressing the illicit drug supply; and identifying specific risk factors for populations using these drugs. Continued surveillance with expanded coverage of additional jurisdictions of the evolving drug overdose epidemic is important to the success of these efforts.

Suspected nonfatal drug overdose ED visits were identified from 29 states* funded through CDC’s Overdose Data to Action program^†^ that submitted data to the National Syndromic Surveillance Program (NSSP).^§^ Querying ED visit data, initial encounter^¶^ unintentional and undetermined intent overdoses were identified using *International Classification of Diseases, Tenth Revision, Clinical Modification* (ICD-10-CM) discharge diagnosis codes for opioids,** cocaine,^††^ amphetamines,^§§^ and benzodiazepines.^¶¶^ Some overdoses involved more than one type of drug, and these were included in calculations for each relevant drug category; thus, categories are not mutually exclusive.*** Data are at the ED-visit level rather than the patient level; therefore, a patient with multiple overdose visits would be included multiple times in analyses.^†††^

The changes in rates of suspected drug overdose per 100,000 ED visits from 2018 to 2019 were calculated overall, by sex, age group, U.S. Census region of the ED facility,^§§§^ and county urbanization level of patient residence.^¶¶¶^ Because syndromic surveillance data were used to examine meaningful changes in suspected overdose-related ED visits and not to estimate numbers of persons with nonfatal drug overdoses, results reported exclude counts and rates. Relative and absolute rate changes**** were calculated from 2018 to 2019 by visit characteristics; chi-squared tests compared 2018 and 2019 rates. Absolute rate changes were included to provide context for relative changes, some of which were based on small numbers of overdoses. Changes presented represent statistically significant findings, unless otherwise specified. Percentages of suspected drug overdose ED visits^††††^ were calculated for specific polydrug combinations to examine the percentages of suspected cocaine-, amphetamine-, and benzodiazepine-involved overdoses that also involved opioids in 2019, overall, and for certain age groups. Chi-squared tests were used for pairwise comparisons between age groups for percentage of overdose ED visits^§§§§^ in 2019. For all analyses, p-values <0.05 were considered statistically significant. Analyses were conducted using SAS (version 9.4; SAS Institute).

From 2018 to 2019, overall relative and absolute rates increased for suspected nonfatal overdoses involving opioids (9.7%; 12.9 per 100,000 ED visits), cocaine (11.0%; 0.7), and amphetamines (18.3%; 1.3); rates decreased for overdoses involving benzodiazepines (−3.0%; −0.5) (Table 1). Relative and absolute rates for overdoses involving opioids increased from 2018 to 2019 among both females (7.1%; 6.0) and males (10.7%; 20.9), as well as all age groups. Cocaine- and amphetamine-involved overdose rates also increased among females (8.5%; 0.3 and 13.1%; 0.6, respectively) and males (12.4%; 1.1 and 20.5%; 2.2, respectively). Relative and absolute rate increases in amphetamine-involved overdoses occurred in all age groups except persons aged 15–24 years; relative and absolute rates of cocaine-involved overdoses increased only among persons aged 35–44 and ≥55 years. Relative and absolute rates of benzodiazepine-involved overdoses decreased among females (−4.4%; −0.7) and among persons aged 15–24 years (−7.3%; −1.7).

**TABLE 1:** Annual change in rates per 100,000 emergency department (ED) visits for suspected unintentional and undetermined intent nonfatal overdoses* involving opioids,^†^ cocaine,^§^ amphetamines,**^¶^** or benzodiazepines,** by sex, age, U.S. Census region, and county urbanization level — 29 states,**^††^** 2018 to 2019

ED patient/visit characteristic	Rate change from 2018 to 2019^§§^							
	Opioids		Cocaine		Amphetamines		Benzodiazepines	
	Relative (%)	Absolute	Relative (%)	Absolute	Relative (%)	Absolute	Relative (%)	Absolute
**All**	**9.7^¶¶^**	**12.9^¶¶^**	**11.0^¶¶^**	**0.7^¶¶^**	**18.3^¶¶^**	**1.3^¶¶^**	**−3.0^¶¶^**	**−0.5^¶¶^**
**Sex**								
Female	7.1^¶¶^	6.0^¶¶^	8.5^¶¶^	0.3^¶¶^	13.1^¶¶^	0.6^¶¶^	−4.4^¶¶^	−0.7^¶¶^
Male	10.7^¶¶^	20.9^¶¶^	12.4^¶¶^	1.1^¶¶^	20.5^¶¶^	2.2^¶¶^	−1.3	−0.2
**Age group, yrs**								
15–24	3.7^¶¶^	4.3^¶¶^	−0.4	0.0	4.3	0.4	−7.3^¶¶^	−1.7^¶¶^
25–34	7.8^¶¶^	22.9^¶¶^	2.0	0.2	18.5^¶¶^	2.9^¶¶^	−3.5	−0.8
35–44	15.2^¶¶^	32.9^¶¶^	20.1^¶¶^	1.9^¶¶^	16.4^¶¶^	2.3^¶¶^	−0.8	−0.2
45–54	14.4^¶¶^	23.2^¶¶^	9.8	1.1	35.8^¶¶^	2.5^¶¶^	−5.2	−1.1
≥55	12.9^¶¶^	9.8^¶¶^	26.4^¶¶^	1.1^¶¶^	60.0^¶¶^	0.9^¶¶^	3.3	0.4
U.S. Census region***								
Northeast	0.0	0.0	10.0	0.6	18.9^¶¶^	0.6^¶¶^	−2.1	−0.3
South	16.5^¶¶^	19.2^¶¶^	12.0^¶¶^	1.0^¶¶^	14.3^¶¶^	1.1^¶¶^	−3.3	−0.6
Midwest	8.3^¶¶^	11.8^¶¶^	14.9^¶¶^	0.7^¶¶^	2.2	0.1	−11.2^¶¶^	−1.5^¶¶^
West	11.5^¶¶^	13.5^¶¶^	8.1	0.3	21.2^¶¶^	3.2^¶¶^	−0.8	−0.2
**County urbanization^†††^**								
Urban	13.6^¶¶^	16.9^¶¶^	16.2^¶¶^	1.0^¶¶^	21.7^¶¶^	1.3^¶¶^	−1.0	−0.2
Rural	10.1^¶¶^	6.1^¶¶^	−7.5	−0.3	20.8^¶¶^	1.9^¶¶^	−5.8	−0.8

* Suspected unintentional and undetermined intent nonfatal overdoses identified using *International Classification of Diseases, Tenth Revision, Clinical Modification* (ICD-10-CM) discharge diagnosis codes.

^†^ Nonfatal suspected unintentional and undetermined intent drug overdoses involving opioids are defined by the following ICD-10-CM discharge diagnosis codes: T40.0X1A, T40.0X4A, T40.1X1A, T40.1X4A, T40.2X1A, T40.2X4A, T40.3X1A, T40.3X4A, T40.4X1A, T40.4X4A, T40.601A, T40.604A, T40.691A, or T40.694A.

^§^ Nonfatal suspected unintentional and undetermined intent drug overdoses involving cocaine are defined by the following ICD-10-CM discharge diagnosis codes: T40.5X1A or T40.5X4A.

^¶^ Nonfatal suspected unintentional and undetermined intent drug overdoses involving amphetamines are defined by the following ICD-10-CM discharge diagnosis codes: T43.621A or T43.624A.

** Nonfatal suspected unintentional and undetermined intent drug overdoses involving benzodiazepines are defined by the following ICD-10-CM discharge diagnosis codes: T42.4X1A or T42.4X4A.

^††^ Alabama, Arizona, Arkansas, Colorado, Connecticut, Delaware, Georgia, Illinois, Kansas, Kentucky, Louisiana, Maine, Maryland, Montana, Nevada, New Jersey, New Mexico, North Carolina, Ohio, Oregon, Pennsylvania, Rhode Island, South Carolina, Tennessee, Utah, Virginia, Washington, West Virginia, and Wisconsin.

^§§^ Estimates are rounded to the nearest tenth. Because of this rounding, estimates of 0.0 are displayed in the tables. These estimates are rounded down from <0.05 and do not represent an absence of a change in rate.

^¶¶^ Statistically significant change (p<0.05).

*** U.S. Census region coded by location of the facility where emergency department visits occurred using values for hospital state. The Northeast region includes hospitals located in five of nine possible states, the South region includes hospitals located in 12 of 16 possible states (17 including the District of Columbia), the Midwest region includes hospitals located in four of 12 possible states, and the West region includes hospitals located in eight of 13 possible states.

^†††^ County urbanization levels for residence county were determined using the 2013 National Center for Health Statistics Urban-Rural Classification Scheme for Counties (https://www.cdc.gov/nchs/data_access/urban_rural.htm). Urban included large central metro, large fringe metro, medium metro, and small metro and rural included micropolitan and noncore counties.

Among U.S. Census regions, relative and absolute increases in rates of opioid-involved overdoses were observed in the South (16.5%; 19.2), West (11.5%; 13.5), and Midwest (8.3%; 11.8); of amphetamine-involved overdoses in the Northeast (18.9%; 0.6), South (14.3%; 1.1), and West (21.2%; 3.2); and of cocaine-involved overdoses in the South (12.0%; 1.0) and Midwest (14.9%; 0.7). The Midwest experienced the only decline in relative and absolute rate for benzodiazepine-involved overdoses (−11.2%; −1.5). Relative and absolute rates of opioid-involved overdoses increased among persons living in both urban (13.6%; 16.9) and rural counties (10.1%; 6.1), as did rates of amphetamine-involved overdoses (21.7%; 1.3, urban and 20.8%; 1.9, rural).

Changes in rates of polydrug overdoses predominantly comprised those co-involving opioids and amphetamines (37.3% relative increase; 0.4 per 100,000 absolute increase) (Table 2). Relative and absolute rate increases for overdoses co-involving opioids and amphetamines were experienced by both females (32.7%; 0.2) and males (38.3%; 0.6) and all age groups except persons aged 45–54 years. Relative and absolute rate increases were identified in the Northeast (116.3%; 0.4), South (33.3%; 0.4), and West (26.7%; 0.7) Census regions. Relative and absolute increases in rates of overdoses co-involving opioids and amphetamines occurred among persons living in urban counties (54.1%; 0.5).

**TABLE 2:** Annual change in rates per 100,000 emergency department (ED) visits for suspected unintentional and undetermined intent nonfatal overdoses* of cocaine,^†^ amphetamines,^§^ benzodiazepines**^¶^** co-involving opioids,** by sex, age, U.S. Census region, and county urbanization level — 29 states,^††^ 2018 to 2019

ED patient/visit characteristic	Rate change from 2018 to 2019^§§^					
	Opioids and cocaine		Opioids and amphetamines		Opioids and benzodiazepines	
	Relative (%)	Absolute	Relative (%)	Absolute	Relative (%)	Absolute
**All**	**4.4**	**0.1**	**37.3^¶¶^**	**0.4^¶¶^**	**2.6**	**0.1**
**Sex**						
Female	0.6	0.0	32.7^¶¶^	0.2^¶¶^	0.3	0.0
Male	6.2	0.1	38.3^¶¶^	0.6^¶¶^	4.9	0.2
**Age group, yrs**						
15–24	1.6	0.0	50.3^¶¶^	0.5^¶¶^	−8.5	−0.2
25–34	−0.1	0.0	35.5^¶¶^	1.0^¶¶^	14.6	0.6
35–44	22.1^¶¶^	0.6^¶¶^	38.6^¶¶^	0.9^¶¶^	−7.2	−0.3
45–54	−0.9	0.0	26.0	0.3	−7.6	−0.3
≥55	15.3	0.1	66.0^¶¶^	0.2^¶¶^	14.5^¶¶^	0.4^¶¶^
U.S. Census region***						
Northeast	−1.4	0.0	116.3^¶¶^	0.4^¶¶^	5.2	0.1
South	6.1	0.1	33.3^¶¶^	0.4^¶¶^	−0.6	0.0
Midwest	19.2	0.2	21.1	0.1	3.0	0.1
West	−13.7	−0.1	26.7^¶¶^	0.7^¶¶^	2.6	0.1
**County urbanization^†††^**						
Urban	11.3^¶¶^	0.2^¶¶^	54.1^¶¶^	0.5^¶¶^	4.3	0.1
Rural	−26.1	−0.2	15.2	0.2	6.8	0.2

* Suspected unintentional and undetermined intent nonfatal overdoses identified using *International Classification of Diseases, Tenth Revision, Clinical Modification* (ICD-10-CM) discharge diagnosis codes.

^†^ Nonfatal suspected unintentional and undetermined intent drug overdoses involving cocaine are defined by the following ICD-10-CM discharge diagnosis codes: T40.5X1A or T40.5X4A.

^§^ Nonfatal suspected unintentional and undetermined intent drug overdoses involving amphetamines are defined by the following ICD-10-CM discharge diagnosis codes: T43.621A or T43.624A.

^¶^ Nonfatal suspected unintentional and undetermined intent drug overdoses involving benzodiazepines are defined by the following ICD-10-CM discharge diagnosis codes: T42.4X1A or T42.4X4A.

** Nonfatal suspected unintentional and undetermined intent drug overdoses involving opioids are defined by the following ICD-10-CM discharge diagnosis codes: T40.0X1A, T40.0X4A, T40.1X1A, T40.1X4A, T40.2X1A, T40.2X4A, T40.3X1A, T40.3X4A, T40.4X1A, T40.4X4A, T40.601A, T40.604A, T40.691A, or T40.694A.

^††^ Alabama, Arizona, Arkansas, Colorado, Connecticut, Delaware, Georgia, Illinois, Kansas, Kentucky, Louisiana, Maine, Maryland, Montana, Nevada, New Jersey, New Mexico, North Carolina, Ohio, Oregon, Pennsylvania, Rhode Island, South Carolina, Tennessee, Utah, Virginia, Washington, West Virginia, and Wisconsin.

^§§^ Estimates are rounded to the nearest tenth. Because of this rounding, estimates of 0.0 are displayed in the tables. These estimates are rounded down from <0.05 and do not represent an absence of a change in rate.

^¶¶^ Statistically significant change (p<0.05).

*** U.S. Census region coded by location of the facility where emergency department visits occurred using values for hospital state. The Northeast region includes hospitals located in five of nine possible states, the South region includes hospitals located in 12 of 16 possible states (17 including the District of Columbia), the Midwest region includes hospitals located in four of 12 possible states, and the West region includes hospitals located in eight of 13 possible states.

^†††^ County urbanization levels for residence county were determined using the 2013 National Center for Health Statistics Urban-Rural Classification Scheme for Counties (https://www.cdc.gov/nchs/data_access/urban_rural.htm). Urban included large central metro, large fringe metro, medium metro, and small metro and rural included micropolitan and noncore counties.

In 2019, opioids were involved in 40.2% of all suspected drug overdoses treated in EDs, including 28.7%, 56.9%, 49.9%, and 34.6% of overdoses among persons aged 15–24, 25–34, 35–54, and ≥55 years, respectively (Figure). In 2019, 23.6% of overdoses involving cocaine, 17.1% involving amphetamines, and 18.7% involving benzodiazepines also involved opioids. The highest percentages of cocaine- (35.0%), amphetamine- (21.1%), and benzodiazepine-involved (23.6%) overdoses that also involved opioids occurred among persons aged 25–34 years.

**FIGURE:**
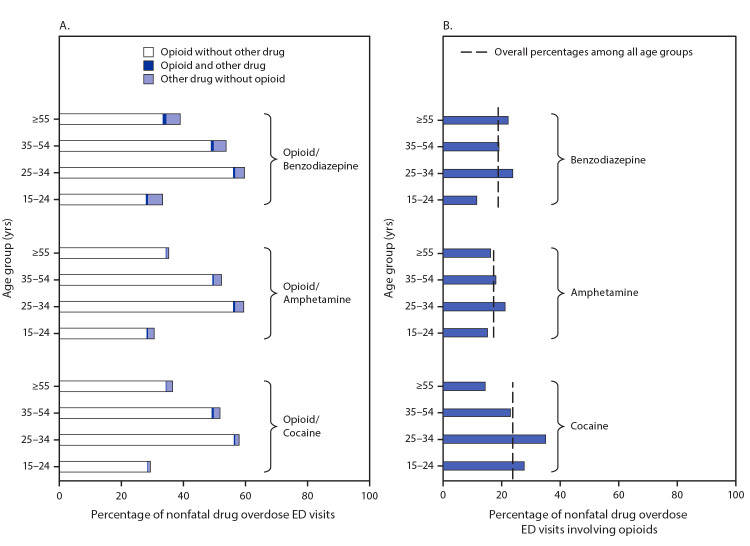
Percentage of nonfatal emergency department (ED) visits for suspected unintentional and undetermined intent nonfatal overdoses* involving combinations of opioids^†^ with and without cocaine,^§^ amphetamines,^¶^ or benzodiazepines** (A)^††^ and percentage of cocaine, amphetamine, and benzodiazepine overdoses involving opioids (B),^§§^ by age group — 29 states,^¶¶^ 2019 * Suspected unintentional and undetermined intent nonfatal overdoses identified using *International Classification of Diseases, Tenth Revision, Clinical Modification* (ICD-10-CM) discharge diagnosis codes. ^†^ Nonfatal suspected unintentional and undetermined intent drug overdoses involving opioids are defined by the following ICD-10-CM discharge diagnosis codes: T40.0X1A, T40.0X4A, T40.1X1A, T40.1X4A, T40.2X1A, T40.2X4A, T40.3X1A, T40.3X4A, T40.4X1A, T40.4X4A, T40.601A, T40.604A, T40.691A, or T40.694A. ^§^ Nonfatal suspected unintentional and undetermined intent drug overdoses involving cocaine are defined by the following ICD-10-CM discharge diagnosis codes: T40.5X1A or T40.5X4A. ^¶^ Nonfatal suspected unintentional and undetermined intent drug overdoses involving amphetamines are defined by the following ICD-10-CM discharge diagnosis codes: T43.621A or T43.624A. ** Nonfatal suspected unintentional and undetermined intent drug overdoses involving benzodiazepines are defined by the following ICD-10-CM discharge diagnosis codes: T42.4X1A or T42.4X4A. ^††^ For overdoses of opioids combined with other drugs, the sum of the bars for "Opioid without other drug" and for "Opioid and other drug" are the percentage totals for opioid-involved overdoses. Opioids were involved in 28.7%, 56.9%. 49.9%, and 34.6% of suspected unintentional and undetermined intent drug overdoses among persons aged 15–24, 25–34, 35–54, and ≥55 years, respectively. ^§§^ For overdoses of cocaine, amphetamines, and benzodiazepines also involving opioid, using pairwise comparisons between age groups, statistically significant (p<0.05) differences include cocaine, persons aged 25–34 years compared with each other age group; amphetamine, persons aged 25–34 years compared with each other age group; benzodiazepines, persons aged 25–34 years compared with persons aged 15–24 and 35–54 years. Overall percentage among all age groups was 18.7% for benzodiazepine, 17.1% for amphetamine, and 23.6% for cocaine-involved overdoses also involving opioids. ^¶¶^ Alabama, Arizona, Arkansas, Colorado, Connecticut, Delaware, Georgia, Illinois, Kansas, Kentucky, Louisiana, Maine, Maryland, Montana, Nevada, New Jersey, New Mexico, North Carolina, Ohio, Oregon, Pennsylvania, Rhode Island, South Carolina, Tennessee, Utah, Virginia, Washington, West Virginia, and Wisconsin.

## Discussion

From 2018 to 2019, rates of suspected nonfatal overdoses involving opioids, cocaine, and amphetamines treated in EDs increased, and those involving benzodiazepines decreased. Despite the decline in nonfatal benzodiazepine-involved overdoses, benzodiazepines were identified in 12.2% of nonfatal overdoses treated in EDs during 2017 (*1*). Benzodiazepines were also one of the most common drug classes identified in overdose deaths,^¶¶¶¶^ likely because of co-use with opioids (*3*). Increases in overdose rates involving other drugs highlight the complicated nature of and challenges associated with addressing the evolving U.S. drug overdose epidemic (*1*). Deaths involving synthetic opioids, primarily illicitly manufactured fentanyl, have been increasing since 2013 (*4*,*5*). In addition, the availability of cocaine and methamphetamine has increased in the United States in recent years, and according to the Drug Enforcement Administration, methamphetamine was the most frequently reported drug among all drug submissions in 2019.*****

Consistent with prior research, opioids constituted a large percentage of drug overdoses overall and were substantially co-involved with stimulant overdoses (*2*). Notably, rates of suspected overdoses co-involving opioids and amphetamines significantly increased from 2018 to 2019, overall, and in both sexes and nearly all age groups. Findings are consistent with previous studies that have highlighted increases in methamphetamine use initiation,^†††††^ co-use between stimulants and opioids (*6*,*7*), nonfatal stimulant-involved overdoses treated in EDs (*8*), and co-involvement of opioids and stimulants in overdose deaths (*9*).

These findings have important programmatic implications regarding the evolving U.S. overdose epidemic. Syndromic surveillance is a critical data source for identifying overdose spikes and clusters to inform deployment of public health and public safety resources. Expanding coverage to include all ED visits in the United States would help further identify certain population characteristics and geographic regions that should be prioritized for prevention, treatment, and response efforts. The increases observed in polydrug overdose rates highlight the complexity of the overdose epidemic and the need to intervene more rapidly before nonfatal polydrug overdoses increase further or result in fatal overdoses.

The findings in this report are subject to at least seven limitations. First, overdose case definitions relied on discharge diagnosis codes, which were missing in 20.3% of ED visits available in NSSP for the 29 states analyzed. Improvements in submission of discharge diagnosis codes might have influenced the changes observed. However, in all included states, visits with valid discharge diagnosis codes increased 5.3% from 2018 to 2019. Second, discharge diagnosis codes might be used inconsistently by hospitals and providers, which could result in misclassification. Third, comprehensive toxicology testing of patients experiencing overdose rarely occurs in overdose ED visits (*10*), which might have underestimated polydrug overdoses. Fourth, hospital participation in NSSP varied across years; thus, results might be related to changes in hospital participation. Fifth, NSSP coverage is not necessarily uniform across or within all states, leading to different levels of coverage by region. Sixth, data are not generalizable beyond states participating in NSSP. Finally, analyses of overdoses stratified by race and ethnicity were not conducted because these data were not available in approximately one third and one half of visits, respectively.

EDs provide an opportunity to intervene and link persons into treatment, harm reduction services, and other community-based programs. Although rates of overdoses co-involving opioids and benzodiazepines were stable from 2018 to 2019, efforts to ensure safe prescribing practices remain critical.^§§§§§^ Provision of naloxone, expanding overdose education to more groups who are at risk, including persons using stimulants, utilizing partnerships between public health and public safety, and an improved understanding of social and structural factors that contribute to overdose are necessary to prevent drug overdoses.

**Summary**

**What is already known about this topic?**

In 2017, a total of 967,615 nonfatal drug overdoses were treated in U.S. emergency departments (EDs); polydrug ED-treated overdoses increased from 2017 to 2018.

**What is added by this report?**

Rates of ED-treated suspected nonfatal drug overdoses involving opioids, cocaine, and amphetamines, and of polydrug overdoses co-involving opioids and amphetamines increased from 2018 to 2019. Rates of suspected benzodiazepine-involved overdoses declined. Opioids were substantially co-involved with cocaine, amphetamine, and benzodiazepine overdoses in 2019; 23.6%, 17.1%, and 18.7% of cocaine-, amphetamine-, and benzodiazepine-involved overdoses, respectively, involved opioids.

**What are the implications for public health practice?**

Opioids have substantial involvement in nonfatal overdoses, including those involving other drugs. Expanding syndromic surveillance to better inform overdose prevention efforts and increasing naloxone provision to persons who use stimulants are essential.

## Footnotes

* Alabama, Arizona, Arkansas, Colorado, Connecticut, Delaware, Georgia, Illinois, Kansas, Kentucky, Louisiana, Maine, Maryland, Montana, Nevada, New Jersey, New Mexico, North Carolina, Ohio, Oregon, Pennsylvania, Rhode Island, South Carolina, Tennessee, Utah, Virginia, Washington, West Virginia, and Wisconsin. Data from these 29 states were analyzed for ED visits from January 1, 2018, to December 31, 2019. The states included had visits with multiple diagnosis codes and <20% change in percentage of ED visits with valid discharge diagnosis codes from 2018 to 2019. The percentage of ED visits with informative discharge diagnosis codes across 29 states was 77.0% and 82.3% in 2018 and 2019, respectively, a change of 5.3%.

† https://www.cdc.gov/drugoverdose/od2a/index.html.

§ https://www.cdc.gov/nssp/documents/NSSP-overview.pdf; https://www.cdc.gov/nssp.

¶
https://www.cdc.gov/injury/wisqars/pdf/ICD-10-CM_External_Cause_Injury_Codes-a.pdf .

** Nonfatal suspected unintentional and undetermined intent drug overdoses involving opioids are defined by the following ICD-10-CM discharge diagnosis codes: T40.0X1A, T40.0X4A, T40.1X1A, T40.1X4A, T40.2X1A, T40.2X4A, T40.3X1A, T40.3X4A, T40.4X1A, T40.4X4A, T40.601A, T40.604A, T40.691A, or T40.694A.

†† Nonfatal suspected unintentional and undetermined intent drug overdoses involving cocaine are defined by the following ICD-10-CM discharge diagnosis codes: T40.5X1A or T40.5X4A.

§§ Nonfatal suspected unintentional and undetermined intent drug overdoses involving amphetamines are defined by the following ICD-10-CM discharge diagnosis codes: T43.621A or T43.624A. Amphetamines are a specific stimulant drug class, distinct from cocaine, that encompass legal prescription medications (e.g., Adderall) and illicit drugs (e.g., methamphetamine). https://www.dea.gov/sites/default/files/drug_of_abuse.pdf.

¶¶ Nonfatal suspected unintentional and undetermined intent drug overdoses involving benzodiazepines are defined by the following ICD-10-CM discharge diagnosis codes: T42.4X1A or T42.4X4A.

*** As an example, an overdose co-involving opioid and cocaine would be included in both the opioid and cocaine change estimates as well as the estimates for polydrug overdoses involving both opioid and cocaine.

††† The unit of analysis was ED visits, not individual patients, and the absence of unique patient identifiers prevents linking ED visits across individual patients to determine the proportions treated in the ED during a single visit versus multiple visits. As an example, a patient treated for nonfatal overdoses in June 2018, October 2018, and March 2019, will reflect three individual ED visits included in the data, analyzed as distinct ED visits.

§§§ U.S. Census region is coded by state of the facility where emergency department visits occurred. The Northeast region includes hospitals located in five of nine possible states, the South region includes hospitals located in 12 of 16 possible states (17 including the District of Columbia), the Midwest region includes hospitals located in four of 12 possible states, and the West region includes hospitals located in eight of 13 possible states.

¶¶¶ County urbanization levels for patient residence county were determined using the 2013 National Center for Health Statistics Urban-Rural Classification Scheme for Counties (https://www.cdc.gov/nchs/data_access/urban_rural.htm). Urban included large central metro, large fringe metro, medium metro, and small metro and rural included micropolitan and noncore counties.

**** Absolute change is the difference in rates from 2018 to 2019. Relative change is the absolute rate change divided by the 2018 rate, multiplied by 100. Because syndromic surveillance data were used to examine meaningful changes in suspected overdose-related ED visits, and not to calculate prevalence estimates regarding numbers of persons with nonfatal drug overdoses, results reported exclude counts and rates.

††††
https://resources.cste.org/ICD-10-CM/Drug%20Overdose%20Indicator/Drug%20Overdose%20Indicator.pdf .

§§§§ Nonfatal drug overdose visits are classified using ICD-10-CM. ICD-10-CM diagnosis codes for all drugs included codes with T36–T50 with a sixth character of 1 or 4 (exceptions for T36.9, T37.9, T39.9, T41.4, T42.7, T43.9, T45.9, T47.9, and T49.9, which were included if the code had a fifth character of 1 or 4). Only codes with a seventh character of “A” (initial encounter) were included.

¶¶¶¶ https://www.cdc.gov/nchs/data/nvsr/nvsr68/nvsr68_12-508.pdf.

***** https://www.nflis.deadiversion.usdoj.gov/DesktopModules/ReportDownloads/Reports/13408NFLISDrugMidYear2019.pdf; https://www.dea.gov/sites/default/files/2020-01/2019-NDTA-final-01-14-2020_Low_Web-DIR-007-20_2019.pdf.

††††† https://www.cdc.gov/drugoverdose/pdf/pubs/2019-cdc-drug-surveillance-report.pdf.

§§§§§ https://www.cdc.gov/drugoverdose/prescribing/guideline.html.
